# Predicting food insecurity in a pediatric population using the electronic health record

**DOI:** 10.1017/cts.2024.645

**Published:** 2024-10-28

**Authors:** Joseph Rigdon, Kimberly Montez, Deepak Palakshappa, Callie Brown, Stephen M. Downs, Laurie W. Albertini, Alysha Taxter

**Affiliations:** 1 Department of Biostatistics and Data Science, Wake Forest School of Medicine, Winston-Salem, USA; 2 Department of Pediatrics, Wake Forest School of Medicine, Winston-Salem, USA; 3 Maya Angelou Center for Health Equity, Wake Forest School of Medicine, Winston-Salem, USA; 4 Department of Epidemiology and Prevention, Wake Forest School of Medicine, Winston-Salem, USA; 5 Center for Healthcare Innovation, Wake Forest School of Medicine, Winston-Salem, USA; 6 Department of Internal Medicine, Wake Forest School of Medicine, Winston-Salem, USA; 7 Center for Biomedical Informatics, Wake Forest School of Medicine, Winston-Salem, USA; 8 Department of Pediatric Rheumatology, Nationwide Children’s Hospital, Columbus, USA; 9 Department of Clinical Informatics, Nationwide Children’s Hospital, Columbus, USA

**Keywords:** Food insecurity, pediatrics, prediction modeling, machine learning, electronic health record

## Abstract

**Introduction:**

More than 5 million children in the United States experience food insecurity (FI), yet little guidance exists regarding screening for FI. A prediction model of FI could be useful for healthcare systems and practices working to identify and address children with FI. Our objective was to predict FI using demographic, geographic, medical, and historic unmet health-related social needs data available within most electronic health records.

**Methods:**

This was a retrospective longitudinal cohort study of children evaluated in an academic pediatric primary care clinic and screened at least once for FI between January 2017 and August 2021. American Community Survey Data provided additional insight into neighborhood-level information such as home ownership and poverty level. Household FI was screened using two validated questions. Various combinations of predictor variables and modeling approaches, including logistic regression, random forest, and gradient-boosted machine, were used to build and validate prediction models.

**Results:**

A total of 25,214 encounters from 8521 unique patients were included, with FI present in 3820 (15%) encounters. Logistic regression with a 12-month look-back using census block group neighborhood variables showed the best performance in the test set (C-statistic 0.70, positive predictive value 0.92), had superior C-statistics to both random forest (0.65, *p* < 0.01) and gradient boosted machine (0.68, *p* = 0.01), and showed the best calibration. Results were nearly unchanged when coding missing data as a category.

**Conclusions:**

Although our models could predict FI, further work is needed to develop a more robust prediction model for pediatric FI.

## Introduction

More than 13.5 million households and 33 million people experience food insecurity (FI), including more than 5 million children in the United States [1]. Notably, FI, or the reduced quality, variety, desirability of diet, or reduced food intake, disproportionately affects households with children under the age of 6 years, headed by Black and Hispanic persons, and headed by a single woman [1]. FI is associated with prematurity; chronic illnesses including asthma, depression, and obesity; missed routine medical care including immunizations; and higher emergency department utilization and hospitalizations [2–4]. Additionally, FI is associated with 20% greater health care expenditures among families with FI compared to families without FI [5].

Given the prevalence and association with poor outcomes, an increasing number of health systems and health insurers are investing in strategies to identify and address FI to improve patient care and population health. Although many healthcare systems are screening for FI, it is not yet universal and little guidance exists regarding the optimal frequency or clinical department, so predicting FI could be useful for healthcare systems and practices that are not yet screening. Additionally, if practices are only screening at well visits, given the transient nature of FI [6], FI may be missed at acute visits, especially among families that are not attending well visits. Furthermore, families may choose not to disclose FI for many reasons, including screening fatigue, shame or stigma, fear of consequences for reporting FI, or perception of need [6–9], so predicting who is at risk of FI and offering resources rather than screening may be useful.

Although there is increasing ability to leverage machine learning techniques using the electronic health record (EHR) to earlier identify diseases and to prevent adverse outcomes [10,11], it is unknown how to best utilize such models to predict which patients are at highest risk of FI to offer resources and interventions. Studies show it is possible to routinely screen for FI during routine clinical care in emergency room, primary care, and subspecialty care settings [12,13]. Given the rich data contained within EHR, including demographics, medical histories, and historic patient-reported information, including unmet health-related social needs, these data could be valuable resources to predict FI. However, such prediction models are not yet validated or integrated into the EHR to guide clinical decision support and patient care.

This study aims to develop a machine learning algorithm to predict families that will report FI using demographic, geographic, medical, and unmet health-related social needs data that are typically available within most EHRs.

## Materials and methods

### Setting

We conducted a retrospective longitudinal cohort study conducted at Atrium Health Wake Forest Baptist between January 2017 and June 2021. This study was approved by the Wake Forest University Health Sciences Institutional Review Board (IRB00071608).

### Study population

We included all patients in one academic pediatric primary care clinic with patients who were screened at least once for FI between January 2017 and August 2021. Encounters were included if FI was screened for and documented in the EHR (*N* = 25,214) and were excluded if FI was not screened for or documented in the EHR (*N* = 3,296). The clinic is in an urban neighborhood and serves a predominantly racial and ethnic minority population of which more than 90% are covered by Medicaid insurance. The medical system is also part of a tertiary care hospital, has multiple board-certified pediatric subspecialties, a pediatric emergency department, and serves the majority of children across western North Carolina.

### Measurement of FI

The clinic screens all patients for FI during routine clinical care, including well-child and acute visits. This screening occurs on paper in English or Spanish and is verbally asked through a certified interpreter if the parent/caregiver speaks another language or is unable to read. Household FI is screened using two validated questions, “In the past year, did you worry that your food would run out before you got money or Food Stamps to buy more?” and “In the past year, did the food you bought just not last and you didn’t have money to get more?” with dichotomous response options (yes or no) [14]. An affirmative response to either question is considered a positive screen. These questions are recommended by the American Academy of Pediatrics [15]. At the time of a clinic visit, the provider discusses the screening results with the family and enters the results into discrete data fields within the EHR. When a patient has a positive screen, the provider asks the family if they would like a bag of food from the clinic’s food pharmacy, referral to a food navigator, connection with federal nutrition programs, and/or a list of community resources.

### Demographics

Patient’s age, sex, race, and ethnicity at the time of the visit were extracted from the EHR. Race and ethnicity, which were self-reported by parents/caregivers and recorded in the EHR, were combined into one variable and categorized as non-Hispanic White, non-Hispanic Black, Hispanic, or other (includes American Indian, Alaska Native, Asian, Native Hawaiian, Other Pacific Islander, Other, Patient Refused, Unknown).

### Other Unmet Health-Related Social Needs Screening

As part of routine clinical care, the clinic also screens for other unmet health-related social needs over the prior 12 months including intimate partner violence, risk of homelessness, transportation difficulties, legal needs, caregiver coping concerns, and caregiver substance use. These needs are screened alongside FI. All screening results and resources provided are documented within the EHR (Supplementary Table 1).

### Chronic conditions and healthcare Utilization

All problem lists and encounter diagnoses from visits occurring at the primary care clinic and any department across the medical center since 2012, when our EHR was adopted, were included. Diagnoses data included in the problem list or encounter diagnoses based on the respective International Classification of Diseases 10th Revision (ICD-10), were extracted, including attention-deficit hyperactivity disorder (ADHD), failure to gain weight, hypertension, asthma, eczema, prematurity, depression, and anxiety. Emergency department visits and hospitalizations were also extracted.

### Anthropometrics

Children’s height and weight are recorded as standard of care at each clinic visit, and body mass index (BMI) percentiles were calculated from the encounter at which FI was measured. Underweight was defined as BMI of <5th percentile. Healthy weight was defined as BMI between ≥ 5 to<85th percentile. Overweight and obesity were defined as BMI of ≥ 85 to <95th, and ≥ 95th percentile, respectively.

### Neighborhood information

We used the US Census Bureau’s 2010 American Community Survey (ACS) 5-year estimates to identify neighborhood information. The ACS includes neighborhood-level information at both the zip code level and the census block level including income, poverty, employment, and home ownership estimates [16]. Zip code predictors included total population count, number of housing units, median age, percentage 25 and older who had a high school education or less, median income, percentage of households with incomes under 100 percent of the federal poverty level in last 12 months, percentage of households where the person who lives in the household owns the house, percentage 16 years of age and older who work and commute 60 minutes or more to work in an area, percentage 16 years of age and older who are unemployed, percent minority, percentage of the area considered to be urban, and percentage of the area considered to be rural. Census block predictors included the same variables as zip code but measured at the smaller geographic level of census block. The home address of all patients in the health system is automatically geocoded by an automated system through the Wake Forest Clinical and Translational Science Institute’s Translational Data Warehouse. We merged geocode data from the EHR with zip code level and census block level data from the ACS.

### Statistical analysis

The analysis cohort was created by merging the above data sources (demographics, anthropometrics, social needs, chronic conditions, and neighborhood information) by encounter number. The analysis cohort was randomly split at the patient level into 80% training set and 20% testing/validation set. Each patient could contribute more than one record to the analysis cohort. Various combinations of predictor variables and modeling approaches were used to train and validate prediction models. Demographic predictor variables included age in years, female sex, Black race, Hispanic ethnicity, other race and ethnicity (defined as non-black, non-Hispanic, and/or nonwhite), and BMI percentile. Twelve-month “look back” indicator variables included previous FI, intimate partner violence, risk of homelessness, transportation difficulties, legal needs, caregiver coping concerns, caregiver substance use, overweight/obesity, ADHD, failure to gain weight, hypertension, asthma, eczema, prematurity, depression, anxiety, hospitalizations, and emergency department visits. The look-back variables were initially coded as absent and were recoded as present if they appeared within the look-back window. The look-back window was expanded for the same set of variables to 18 and 24 months. Six different iterations of each logistic regression, random forest, and gradient-boosted model, respectively, were developed. The modeling approaches were defined by ACS census block variables with a 12, 18, or 24-month clinical data look-back period, or ACS zip code variables using 12, 18, or 24-month clinical data look-back period. All models included demographics.

Modeling approaches included logistic regression, random forest, and gradient-boosted machine. Default values of hyperparameters were used for random forest (500 trees, number of variables to split at each node equal to the rounded down square root of the number of predictors, minimum node size of 1) and gradient boosted machine (learning rate of 0.3, maximum tree depth of 6) [17–20]. No variable selection was performed, as all variables considered are readily available in the EHR. All models were built on the training set using 10-fold cross-validation to estimate out-of-sample performance for the metrics of accuracy, area under the curve (C-statistic), precision-recall area under the curve, sensitivity, specificity, positive predictive value (PPV), and negative predictive value (NPV). In the 10-fold cross-validation, each patient’s observations were uniquely mapped to only one of the folds to prevent overly optimistic estimates of performance. Final models from the training set were then evaluated on the test set. Test set evaluations included measures of discrimination and calibration. Model discrimination was measured using the C-statistic and compared between models using DeLong’s test [21,22]. Model calibration was measured using the Hosmer–Lemeshow slope statistic [23]. The Hosmer–Lemeshow statistic breaks the data into deciles of predicted FI and examines the relationship by decile between average predicted FI and observed rate of FI. A slope of 1 for a line connecting the deciles indicates a well-calibrated model.

Missing data were addressed in two ways. First, a single imputation was employed to fill in all missing data, using multiple imputations by chained equations [24]. As a sensitivity analysis, missing data were coded as a category [25]. Continuous variables were summarized using tertiles, with a fourth level added to reflect missing data. Categorical variables included an extra level to reflect missing, e.g., sex was coded as male, female, or missing. Additional sensitivity analyses included using only data from before the onset of the COVID-19 pandemic (visits occurring before 3/11/2020) and removing previous FI as a predictor from the model.

All analyses were conducted using R version 4.3.2 [26]. Machine learning prediction models were trained and validated using the R package “mlr3” [27]. All models were built and described using the principles outlined in the Transparent reporting of a multivariable prediction model for individual prognosis or diagnosis (TRIPOD) statement [28] (Supplementary Table 2).

## Results

A total of 25,214 encounters from 8521 unique patients collected from 1/3/2017 to 8/2/2021 met inclusion criteria for this study. The number of encounters per patient ranged from 1–12 with a median of 2 (IQR: 1, 4). Patients were partitioned into 80% training (N = 6816 patients with 20,171 encounters) and 20% test/validation (N = 1705 patients with 5043 encounters). The median age was 3.17 years (IQR: 0.75, 9.17), and the sample was 52% male, 22.4% Black, and 62.0% Hispanic. The median BMI percentile was 76 (IQR 47.15, 93.77), and 26.2% of the sample was under 100% of the federal poverty level in last 12 months. Training and test sets showed similar demographics (Tables 1–3). Overall, 3820 (15.2%) encounters reported FI, with similar proportions of FI in the training (N = 3054, 15.1%) and test (N = 766, 15.4%) sets.

**Table 1.** Demographic predictor variables in model training and test data sets

	Training	Test	Total
	*n* = 20,233	*n* = 4981	*n* = 25,214
**Age (years)**	5.2 (±5.1)	5.2 (±5.0)	5.2 (±5.1)
**Gender**			
Female	9,619 (47.5%)	2,478 (49.7%)	12,097 (48.0%)
Male	10,614 (52.5%)	2,503 (50.3%)	13,117 (52.0%)
**Race/ethnicity**			
Black	4,495 (22.2%)	1,147 (23.0%)	5,642 (22.4%)
White	921 (4.6%)	198 (4.0%)	1,119 (4.4%)
Other	2,310 (11.4%)	514 (10.3%)	2,824 (11.2%)
Hispanic	12,507 (61.8%)	3,122 (62.7%)	15,629 (62.0%)
**BMI percentile**	67.7 (±28.9)	69.5 (±27.6)	68.0 (±28.7)
Missing	93 (0.5%)	25 (0.5%)	118 (0.5%)
**Total population block group**	1585.0 (±719.3)	1582.9 (±728.7)	1584.6 (±721.2)
Missing	1,525 (7.5%)	331 (6.6%)	1,856 (7.4%)
**Total population ZIP**	40,108.8 (±11337.8)	39,882.3 (±11136.7)	40,064.0 (±11298.5)
Missing	56 (0.3%)	13 (0.3%)	69 (0.3%)
**Housing units block group**	671.3 (±260.0)	659.8 (±254.4)	669.0 (±258.9)
Missing	1,525 (7.5%)	331 (6.6%)	1,856 (7.4%)
**Housing units ZIP**	17,571.7 (±4511.9)	17,481.9 (±4431.5)	17,554.0 (±4496.2)
Missing	56 (0.3%)	13 (0.3%)	69 (0.3%)
**Median age block group**	35.4 (±9.6)	35.4 (±9.4)	35.4 (±9.6)
Missing	1,525 (7.5%)	331 (6.6%)	1,856 (7.4%)
**Median age ZIP**	37.4 (±3.0)	37.3 (±3.0)	37.4 (±3.0)
Missing	56 (0.3%)	13 (0.3%)	69 (0.3%)
**% >25yo with HS degree block group**	46.3 (±15.3)	46.2 (±15.8)	46.2 (±15.4)
Missing	1,525 (7.5%)	331 (6.6%)	1,856 (7.4%)
**% >25yo with HS degree ZIP**	38.7 (±9.5)	38.7 (±9.6)	38.7 (±9.5)
Missing	56 (0.3%)	13 (0.3%)	69 (0.3%)
**Median income block group**	40,677.7 (±18623.7)	41,355.4 (±20400.1)	40,811.6 (±18989.2)
Missing	3,274 (16.2%)	806 (16.2%)	4,080 (16.2%)
**Median income ZIP**	46,114.0 (±10492.6)	46,023.6 (±10716.5)	46,096.1 (±10537.1)
Missing	56 (0.3%)	13 (0.3%)	69 (0.3%)
**% below poverty line block group**	26.2 (±16.3)	26.4 (±16.8)	26.2 (±16.4)
Missing	1,525 (7.5%)	331 (6.6%)	1,856 (7.4%)
**% below poverty line ZIP**	19.1 (±6.4)	19.2 (±6.4)	19.1 (±6.4)
Missing	56 (0.3%)	13 (0.3%)	69 (0.3%)
**% home ownership block group**	46.9 (±26.7)	47.4 (±27.4)	47.0 (±26.9)
Missing	1,525 (7.5%)	331 (6.6%)	1,856 (7.4%)
**% home ownership ZIP**	58.8 (±12.3)	58.4 (±12.4)	58.7 (±12.3)
Missing	56 (0.3%)	13 (0.3%)	69 (0.3%)
**% >16yo commuting 60 mins block group**	5.0 (±6.8)	5.2 (±7.2)	5.0 (±6.9)
Missing	1,525 (7.5%)	331 (6.6%)	1,856 (7.4%)
**% >16yo commuting 60 mins ZIP**	4.3 (±1.2)	4.3 (±1.2)	4.3 (±1.2)
Missing	56 (0.3%)	13 (0.3%)	69 (0.3%)
**% >16yo unemployed block group**	3.6 (±3.3)	3.5 (±3.2)	3.6 (±3.3)
Missing	1,525 (7.5%)	331 (6.6%)	1,856 (7.4%)
**% >16yo unemployed ZIP**	3.6 (±1.2)	3.7 (±1.2)	3.6 (±1.2)
Missing	56 (0.3%)	13 (0.3%)	69 (0.3%)
**% minority block group**	55.8 (±24.8)	57.4 (±24.8)	56.1 (±24.8)
Missing	1,525 (7.5%)	331 (6.6%)	1,856 (7.4%)
**% minority ZIP**	45.1 (±16.5)	45.6 (±16.5)	45.2 (±16.5)
Missing	56 (0.3%)	13 (0.3%)	69 (0.3%)
**% area urban block group**	91.0 (±15.6)	91.6 (±14.9)	91.1 (±15.5)
Missing	48 (0.2%)	11 (0.2%)	59 (0.2%)
**% area urban ZIP**	91.9 (±14.1)	92.6 (±13.0)	92.0 (±13.9)
Missing	55 (0.3%)	13 (0.3%)	68 (0.3%)
**% area rural block group**	9.0 (±15.6)	8.4 (±14.9)	8.9 (±15.5)
Missing	48 (0.2%)	11 (0.2%)	59 (0.2%)
**% area rural block ZIP**	8.1 (±14.1)	7.4 (±13.0)	8.0 (±13.9)
Missing	55 (0.3%)	13 (0.3%)	68 (0.3%)

Legend: N (%) or mean (SD) are reported. BMI = body mass index. HS = high school. Mins = minutes. SD = standard deviation. ZIP = zip code. yo = year old.

**Table 2.** Social determinants of health predictor variables in model training and test data sets

	Training	Test	Total
	*n* = 20,233	*n* = 4981	*n* = 25,214
**FI last 12 months**			
No	6,213 (30.7%)	1,489 (29.9%)	7,702 (30.5%)
Yes	1,174 (5.8%)	258 (5.2%)	1,432 (5.7%)
Missing	12,846 (63.5%)	3,234 (64.9%)	16,080 (63.8%)
**FI last 18 months**			
No	8,802 (43.5%)	2,158 (43.3%)	10,960 (43.5%)
Yes	1,625 (8.0%)	389 (7.8%)	2,014 (8.0%)
Missing	9,806 (48.5%)	2,434 (48.9%)	12,240 (48.5%)
**FI last 24 months**			
No	9,493 (46.9%)	2,322 (46.6%)	11,815 (46.9%)
Yes	1,824 (9.0%)	443 (8.9%)	2,267 (9.0%)
Missing	8,916 (44.1%)	2,216 (44.5%)	11,132 (44.2%)
**Intimate partner violence last 12 months**			
No	7,341 (36.3%)	1,734 (34.8%)	9,075 (36.0%)
Yes	46 (0.2%)	13 (0.3%)	59 (0.2%)
Missing	12,846 (63.5%)	3,234 (64.9%)	16,080 (63.8%)
**Intimate partner violence last 18 months**			
No	10,357 (51.2%)	2,532 (50.8%)	12,889 (51.1%)
Yes	70 (0.3%)	15 (0.3%)	85 (0.3%)
Missing	9,806 (48.5%)	2,434 (48.9%)	12,240 (48.5%)
**Intimate partner violence last 24 months**			
No	11,235 (55.5%)	2,747 (55.1%)	13,982 (55.5%)
Yes	82 (0.4%)	18 (0.4%)	100 (0.4%)
Missing	8,916 (44.1%)	2,216 (44.5%)	11,132 (44.2%)
**Homeless last 12 months**			
No	7,266 (35.9%)	1,713 (34.4%)	8,979 (35.6%)
Yes	121 (0.6%)	34 (0.7%)	155 (0.6%)
Missing	12,846 (63.5%)	3,234 (64.9%)	16,080 (63.8%)
**Homeless last 18 months**			
No	10,248 (50.6%)	2,484 (49.9%)	12,732 (50.5%)
Yes	179 (0.9%)	63 (1.3%)	242 (1.0%)
Missing	9,806 (48.5%)	2,434 (48.9%)	12,240 (48.5%)
**Homeless last 24 months**			
No	11,126 (55.0%)	2,702 (54.2%)	13,828 (54.8%)
Yes	191 (0.9%)	63 (1.3%)	254 (1.0%)
Missing	8,916 (44.1%)	2,216 (44.5%)	11,132 (44.2%)
**Transportation difficulties last 12 months**			
No	6,924 (34.2%)	1,663 (33.4%)	8,587 (34.1%)
Yes	463 (2.3%)	84 (1.7%)	547 (2.2%)
Missing	12,846 (63.5%)	3,234 (64.9%)	16,080 (63.8%)
**Transportation difficulties last 18 months**			
No	9,803 (48.5%)	2,432 (48.8%)	12,235 (48.5%)
Yes	624 (3.1%)	115 (2.3%)	739 (2.9%)
Missing	9,806 (48.5%)	2,434 (48.9%)	12,240 (48.5%)
**Transportation difficulties last 24 months**			
No	10,621 (52.5%)	2,634 (52.9%)	13,255 (52.6%)
Yes	696 (3.4%)	131 (2.6%)	827 (3.3%)
Missing	8,916 (44.1%)	2,216 (44.5%)	11,132 (44.2%)
**Legal issues last 12 months**			
No	7,284 (36.0%)	1,720 (34.5%)	9,004 (35.7%)
Yes	103 (0.5%)	27 (0.5%)	130 (0.5%)
Missing	12,846 (63.5%)	3,234 (64.9%)	16,080 (63.8%)
**Legal issues last 18 months**			
No	10,253 (50.7%)	2,507 (50.3%)	12,760 (50.6%)
Yes	174 (0.9%)	40 (0.8%)	214 (0.8%)
Missing	9,806 (48.5%)	2,434 (48.9%)	12,240 (48.5%)
**Legal issues last 24 months**			
No	11,129 (55.0%)	2,722 (54.6%)	13,851 (54.9%)
Yes	188 (0.9%)	43 (0.9%)	231 (0.9%)
Missing	8,916 (44.1%)	2,216 (44.5%)	11,132 (44.2%)
**Caregiver coping concerns last 12 months**			
No	20,203 (99.9%)	4,978 (99.9%)	25,181 (99.9%)
Yes	30 (0.1%)	3 (0.1%)	33 (0.1%)
**Caregiver coping concerns last 18 months**			
No	20,196 (99.8%)	4,978 (99.9%)	25,174 (99.8%)
Yes	37 (0.2%)	3 (0.1%)	40 (0.2%)
**Caregiver coping concerns last 24 months**			
No	20,194 (99.8%)	4,977 (99.9%)	25,171 (99.8%)
Yes	39 (0.2%)	4 (0.1%)	43 (0.2%)
**Caregiver substance use last 12 months**			
No	6,392 (31.6%)	1,544 (31.0%)	7,936 (31.5%)
Yes	995 (4.9%)	203 (4.1%)	1,198 (4.8%)
Missing	12,846 (63.5%)	3,234 (64.9%)	16,080 (63.8%)
**Caregiver substance use last 18 months**			
No	9,057 (44.8%)	2,265 (45.5%)	11,322 (44.9%)
Yes	1,370 (6.8%)	282 (5.7%)	1,652 (6.6%)
Missing	9,806 (48.5%)	2,434 (48.9%)	12,240 (48.5%)
**Caregiver substance use last 24 months**			
No	9,771 (48.3%)	2,447 (49.1%)	12,218 (48.5%)
Yes	1,546 (7.6%)	318 (6.4%)	1,864 (7.4%)
Missing	8,916 (44.1%)	2,216 (44.5%)	11,132 (44.2%)

Legend: FI = food insecurity.

**Table 3.** ICD-10 predictor variables in model training and test data sets

	Training	Test	Total
	*n* = 20,233	*n* = 4981	*n* = 25,214
**ADHD last 12 months**			
No	19,917 (98.4%)	4,898 (98.3%)	24,815 (98.4%)
Yes	316 (1.6%)	83 (1.7%)	399 (1.6%)
**ADHD last 18 months**			
No	19,839 (98.1%)	4,882 (98.0%)	24,721 (98.0%)
Yes	394 (1.9%)	99 (2.0%)	493 (2.0%)
**ADHD last 24 months**			
No	19,782 (97.8%)	4,868 (97.7%)	24,650 (97.8%)
Yes	451 (2.2%)	113 (2.3%)	564 (2.2%)
**Overweight/obesity last 12 months**			
No	17,652 (87.2%)	4,324 (86.8%)	21,976 (87.2%)
Yes	2,581 (12.8%)	657 (13.2%)	3,238 (12.8%)
**Overweight/obesity last 18 months**			
No	17,226 (85.1%)	4,199 (84.3%)	21,425 (85.0%)
Yes	3,007 (14.9%)	782 (15.7%)	3,789 (15.0%)
**Overweight/obesity last 24 months**			
No	17,034 (84.2%)	4,140 (83.1%)	21,174 (84.0%)
Yes	3,199 (15.8%)	841 (16.9%)	4,040 (16.0%)
**Failure to gain weight last 12 months**			
No	19,629 (97.0%)	4,869 (97.8%)	24,498 (97.2%)
Yes	604 (3.0%)	112 (2.2%)	716 (2.8%)
**Failure to gain weight last 18 months**			
No	19,488 (96.3%)	4,830 (97.0%)	24,318 (96.4%)
Yes	745 (3.7%)	151 (3.0%)	896 (3.6%)
**Failure to gain weight last 24 months**			
No	19,369 (95.7%)	4,809 (96.5%)	24,178 (95.9%)
Yes	864 (4.3%)	172 (3.5%)	1,036 (4.1%)
**Hypertension last 12 months**			
No	20,157 (99.6%)	4,962 (99.6%)	25,119 (99.6%)
Yes	76 (0.4%)	19 (0.4%)	95 (0.4%)
**Hypertension last 18 months**			
No	20,127 (99.5%)	4,957 (99.5%)	25,084 (99.5%)
Yes	106 (0.5%)	24 (0.5%)	130 (0.5%)
**Hypertension last 24 months**			
No	20,112 (99.4%)	4,954 (99.5%)	25,066 (99.4%)
Yes	121 (0.6%)	27 (0.5%)	148 (0.6%)
**Asthma last 12 months**			
No	19,599 (96.9%)	4,828 (96.9%)	24,427 (96.9%)
Yes	634 (3.1%)	153 (3.1%)	787 (3.1%)
**Asthma last 18 months**			
No	19,504 (96.4%)	4,798 (96.3%)	24,302 (96.4%)
Yes	729 (3.6%)	183 (3.7%)	912 (3.6%)
**Asthma last 24 months**			
No	19,435 (96.1%)	4,781 (96.0%)	24,216 (96.0%)
Yes	798 (3.9%)	200 (4.0%)	998 (4.0%)
**Eczema last 12 months**			
No	19,293 (95.4%)	4,759 (95.5%)	24,052 (95.4%)
Yes	940 (4.6%)	222 (4.5%)	1,162 (4.6%)
**Eczema last 18 months**			
No	18,956 (93.7%)	4,687 (94.1%)	23,643 (93.8%)
Yes	1,277 (6.3%)	294 (5.9%)	1,571 (6.2%)
**Eczema last 24 months**			
No	18,712 (92.5%)	4,627 (92.9%)	23,339 (92.6%)
Yes	1,521 (7.5%)	354 (7.1%)	1,875 (7.4%)
**Prematurity last 12 months**			
No	19,480 (96.3%)	4,838 (97.1%)	24,318 (96.4%)
Yes	753 (3.7%)	143 (2.9%)	896 (3.6%)
**Prematurity last 18 months**			
No	19,323 (95.5%)	4,811 (96.6%)	24,134 (95.7%)
Yes	910 (4.5%)	170 (3.4%)	1,080 (4.3%)
**Prematurity last 24 months**			
No	19,241 (95.1%)	4,799 (96.3%)	24,040 (95.3%)
Yes	992 (4.9%)	182 (3.7%)	1,174 (4.7%)
**Anxiety last 12 months**			
No	20,001 (98.9%)	4,925 (98.9%)	24,926 (98.9%)
Yes	232 (1.1%)	56 (1.1%)	288 (1.1%)
**Anxiety last 18 months**			
No	19,934 (98.5%)	4,908 (98.5%)	24,842 (98.5%)
Yes	299 (1.5%)	73 (1.5%)	372 (1.5%)
**Anxiety last 24 months**			
No	19,893 (98.3%)	4,900 (98.4%)	24,793 (98.3%)
Yes	340 (1.7%)	81 (1.6%)	421 (1.7%)
**Hospitalization last 12 months**			
No	19,918 (98.4%)	4,944 (99.3%)	24,862 (98.6%)
Yes	315 (1.6%)	37 (0.7%)	352 (1.4%)
**Hospitalization last 18 months**			
No	19,792 (97.8%)	4,919 (98.8%)	24,711 (98.0%)
Yes	441 (2.2%)	62 (1.2%)	503 (2.0%)
**Hospitalization last 24 months**			
No	19,699 (97.4%)	4,901 (98.4%)	24,600 (97.6%)
Yes	534 (2.6%)	80 (1.6%)	614 (2.4%)
**Emergency department visit last 12 months**			
No	19,700 (97.4%)	4,859 (97.6%)	24,559 (97.4%)
Yes	533 (2.6%)	122 (2.4%)	655 (2.6%)
**Emergency department visit last 18 months**			
No	19,235 (95.1%)	4,725 (94.9%)	23,960 (95.0%)
Yes	998 (4.9%)	256 (5.1%)	1,254 (5.0%)
**Emergency department visit last 24 months**			
No	18,642 (92.1%)	4,589 (92.1%)	23,231 (92.1%)
Yes	1,591 (7.9%)	392 (7.9%)	1,983 (7.9%)

Legend: ADHD = attention deficit and hyperactivity disorder.

Logistic regression with a 12-month look-back using census block group neighborhood variables showed the best performance in the training set (Table 4). This approach had a C-statistic of 0.68 and an accuracy of 0.85. The most important variables in this model (Supplementary Table 3) included previous FI (odds ratio 4.09; *p*-value < .0001), domestic violence (odds ratio 0.24; *p*-value < .0001), failure to gain weight (odds ratio 1.43; *p*-value 0.0007), and prematurity (odds ratio 1.24; *p*-value 0.0264). BMI percentile, age, and income had the highest variable importance in the random forest model, whereas domestic violence, previous FI, and age had the highest variable importance values in the gradient boosting model.

**Table 4.** Results from 10-fold cross-validation on training set, by geographic level (census block vs. zip code) and look-back time (12, 18, and 24 months)

		Census block			ZIP code		
		12	18	24	12	18	24
**Accuracy**	LR	0.8483	0.8505	0.8490	0.8501	0.8512	0.8504
	RF	0.8438	0.8426	0.8423	0.8495	0.8497	0.8495
	GBM	0.8435	0.8430	0.8405	0.8492	0.8498	0.8466
**AUC**	LR	0.6814	0.6714	0.6795	0.6771	0.6665	0.6762
	RF	0.6616	0.6580	0.6565	0.6599	0.6515	0.6599
	GBM	0.6563	0.6591	0.6628	0.6687	0.6643	0.6636
**PR-AUC**	LR	0.3076	0.3031	0.3066	0.3083	0.3036	0.3078
	RF	0.2620	0.2527	0.2542	0.2903	0.2784	0.2862
	GBM	0.2805	0.2713	0.2664	0.2870	0.2903	0.2783
**Sensitivity**	LR	0.0425	0.0399	0.0293	0.0439	0.0371	0.0313
	RF	0.0539	0.0421	0.0451	0.0513	0.0442	0.0442
	GBM	0.0636	0.0750	0.0622	0.0583	0.0617	0.0535
**Specificity**	LR	0.9917	0.9948	0.9949	0.9937	0.9962	0.9964
	RF	0.9851	0.9857	0.9848	0.9917	0.9932	0.9929
	GBM	0.9831	0.9799	0.9797	0.9900	0.9901	0.9879
**PPV**	LR	0.4658	0.5749	0.5168	0.5662	0.6425	0.6171
	RF	0.3940	0.3309	0.3227	0.5307	0.5413	0.5312
	GBM	0.3987	0.4101	0.3494	0.5205	0.5364	0.4438
**NPV**	LR	0.8533	0.8533	0.8519	0.8536	0.8530	0.8523
	RF	0.8536	0.8522	0.8524	0.8544	0.8536	0.8536
	GBM	0.8546	0.8560	0.8541	0.8550	0.8555	0.8541

Legend: AUC = area under the cure. PR-AUC = precision recall- area under the curve. PPV = positive predictive value. NPV = negative predictive value. LR = logistic regression. RF = random forest. GBM = gradient boosted model.

**Figure 1.**
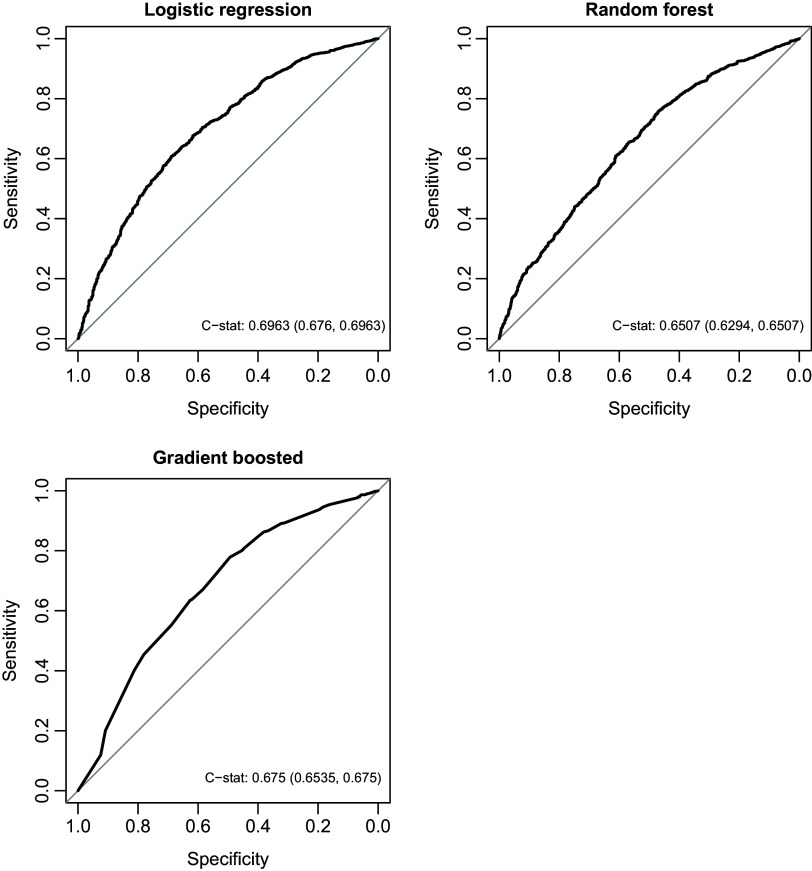
Discrimination statistics (C-statistics) for logistic regression, random forest, and gradient boosted models in test set to predict food insecurity. Legend: C-stat = C-statistic.

**Figure 2.**
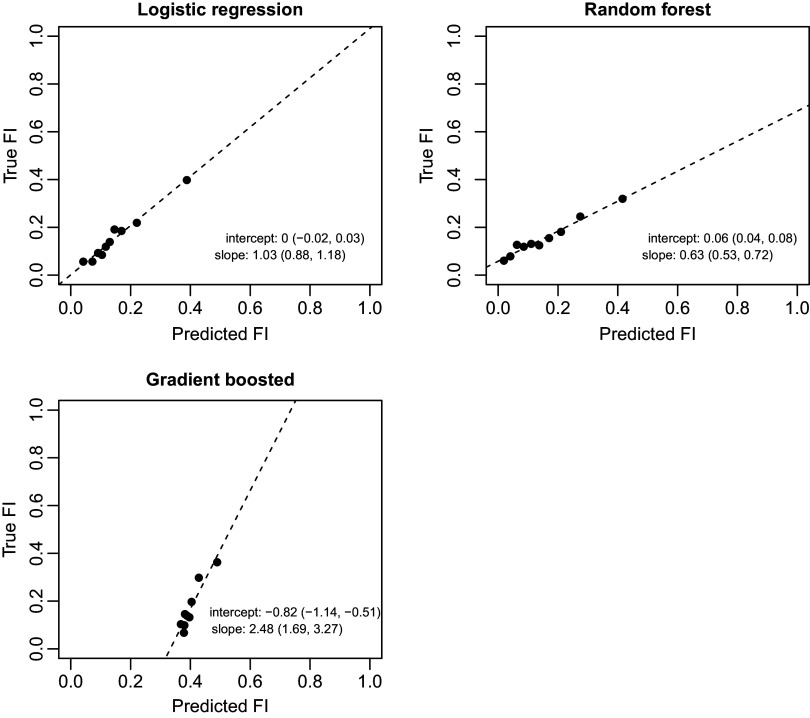
Calibration statistics for logistic regression, random forest, and gradient boosted model in test set to predict food insecurity. Legend: FI = food insecurity.

Logistic regression with 12-month lookback using census block group neighborhood variables performed similarly in the test set (C-statistic 0.70 and accuracy 0.84) and had superior C-statistics to both random forest (0.65, *p* = 0.01) and gradient boosted machine (0.68, *p* < 0.01) (Table 5, Figure 1). Furthermore, this approach showed the best calibration (Figure 2), with a predicted versus true intercept of 0 and 95% CI (-0.02, 0.03) that includes 0, reflecting good calibration, and slope of 1.03 and 95% CI (0.88, 1.18) that includes 1, also reflecting good calibration. Sensitivity and specificity were maximized when predicting FI for all encounters with probability of 0.13 or higher (Supplementary Tables 4 and 5). Notably, at the optimal cutpoint of 0.13, PPV was equal to 0.92.

**Table 5.** Model performance in test set. Predictors chosen are those at the census block level and a 12-month look-back, the best-performing set in Table 3

	Census block + 12 month look back	
**Accuracy**	LR	0.8440
	RF	0.8414
	GBM	0.8412
**AUC**	LR	0.6963
	RF	0.6507
	GBM	0.6750
**PR-AUC**	LR	0.2989
	RF	0.2492
	GBM	0.2799
**Sensitivity**	LR	0.0235
	RF	0.0326
	GBM	0.0601
**Specificity**	LR	0.9931
	RF	0.9884
	GBM	0.9832
**PPV**	LR	0.3830
	RF	0.3378
	GBM	0.3932
**NPV**	LR	0.8484
	RF	0.8490
	GBM	0.8520

Legend: AUC = area under the cure. PR-AUC = precision recall- area under the curve. PPV = positive predictive value. NPV = negative predictive value. LR = logistic regression. RF = random forest. GBM = gradient boosted model.

Model Performance was similar when treating missing data as a category rather than imputing (Supplementary Tables 6 and 7). With missing data coded as a category, the logistic regression with a 24-month look back using census block group posted a C-statistic of 0.70 and an accuracy of 0.85, showing nearly identical performance to handling missing data using a single imputation.

Two additional sensitivity analyses were performed. First, only data from before the onset of the COVID-19 pandemic (before 3/11/2020) were included in model training and testing. In this analysis, there were 14,712 encounters from 6180 patients in the training set and 3573 encounters from 1538 patients in the test set. Again using 12-month lookback data with census block group neighborhood variables, logistic regression had a C-statistic of 0.69 in the test set, indicating similar performance to the model built on the full data set. A second sensitivity analysis removed previous FI as a predictor from the model. Doing so had a large impact on the predictive ability of the model, reducing the C-statistic in the test set to 0.63.

## Discussion

This is one of the first studies to utilize machine learning to predict pediatric FI. Our study shows logistic regression modeling with a 12-month look-back using census block group neighborhood variables, demographics, unmet health-related social needs, and clinical data from the EHR showed the best performance in both the training and validation sets to predict FI with a C-statistic of 0.70. Interestingly, when missing data were encoded as a categorical variable rather than imputed, the model exhibited similar performance, with a C-statistic of 0.70.

Notably, despite evaluating more than 80 variables, leveraging historical longitudinal data, routine health-related social screening at well and acute visits, and including a 12-month look-back period, our most robust model did not perform as well as expected with a C-statistic of only 0.70. This is particularly noteworthy in a pediatric population, when we first start evaluating patients at birth, and provide routine well-child visits every 2–3 months for the first two years. It is possible that prediction modeling of FI in families that have infants and toddlers is less accurate. Similarly, given that our clinic serves a racial and ethnic minority population, it is also possible that families in our clinic under-report FI due to fear of consequences, or perception of need [6–9]. Comparable clinics have a wide positive FI screening rates ranging from 10% to more than 70% [29–31]. This study also highlights that despite advanced modeling, there are unmeasured variables, and screening for FI remains important given the lack of effective predictive models. This is in accordance with recent guidance from regulatory agencies for screening health-related social needs, including FI, to reduce disparities [32–34].

Given our model’s performance, it is also possible that expanded FI screening to other departments outside of our clinic may create a more robust model. Patients and families with unmet health-related social needs seek acute medical care more frequently than those without unmet needs [35,36], so it is possible families are not seeing their primary care provider, which was necessary to be included in our study. Likewise, our clinic has access to federal and community-based nutrition supports, such as the Special Supplemental Nutrition Program for Women, Infants and Children, Supplemental Nutrition Assistance Programs, food pantries, and other hunger relief programs embedded within our clinic and clinical workflows, it is possible that some FI needs were being partially addressed. Similarly, although there are brief, validated surveys for FI that could be used universally, this simply adds to the large amount of screening recommended but not being done, including depression, anxiety, homelessness, domestic violence, etc. Rather than add one more screen that may supplant another, we propose targeting the highest-risk patients to address FI using a statistical model that runs in the background and does not require doctor-patient interaction unless patients meet a threshold of likely FI.

Strengths of this study include that it leverages the EHR and community data, which are rich data sources containing demographic, unmet health-related social needs, and health information. This study is an example of the real-world applicability of integrating social determinants of health and the electronic health record to enable predictive models for social needs screening and intervention. We also had a large sample size in an urban pediatric health center, which is an ideal location to screen for FI and assist with addressing unmet health-related social needs.

A limitation of the study is that we did not address potential correlation between multiple encounters within the same person. Potential approaches could include longitudinal modeling [37,38] or creating a number of encounter variables and including them in the model [39]. Similarly, other models to predict FI include variables on education and school performance, patient substance use, and family relationships; we attempted to address these by including parental substance use screening but did not have access to school or family data outside of the ACS data. It is also possible ACS survey has nonresponse bias; however, the US Bureau takes steps in sampling and weights to account for nonresponse bias and to improve the quality of data. Our data are retrospective; there could be selection bias in who was screened for FI, but we standardized clinic procedures to collect these data [40]. Lastly, FI is commonly measured at the family level; however, we were unable to accurately identify sibling and family linkages, and whether or not children were split between households at different encounters.

## Conclusion

This is one of the first pediatric studies leveraging machine learning to predict FI. Although such models can be integrated into the EHR to predict FI, guide clinical decision support, and streamline workflows to address FI in a pediatric population, further work is needed to develop a more robust prediction model. Future considerations include more widespread integration of FI screening to assist with model development.

## Supporting information

Rigdon et al. supplementary material Rigdon et al. supplementary material

## Supplementary material

The supplementary material for this article can be found at https://doi.org/10.1017/cts.2024.645.
