# Supplementary material for: Predicting food insecurity in a pediatric population using the electronic health record
Source: J Clin Transl Sci. 2024 Oct 28;8(1):e195. doi: 10.1017/cts.2024.645 (PMC11626594; doi:10.1017/cts.2024.645)
Supplement: Rigdon et al. supplementary material [file S2059866124006459sup001.docx]

**Supplementary Appendix**

**Supplementary Table 1:** Patient-Reported Social Needs Screening Questionnaire

| 1. In the past year, did you worry that your food would run out before you got money or Food Stamps to buy more?  *En el último año, ¿estuvo preocupada(o) que se le acabara la comida antes de recibir dinero o cupones para comprar más alimentos?* | □ Yes/Sí □ No |
| --- | --- |
| 2. In the past year, did the food you bought just not last and you didn’t have money to get more?  *En el último año, ¿Se quedó sin comida, y no tuvo dinero para comprar más?* | □ Yes/Sí □ No |
| 3. In the past year, have you been afraid of your partner?  *En el último año, ¿alguna vez tuvo miedo de su pareja?* | □ Yes/Sí □ No |
| 4. In the past year, have you ever felt unsafe in your home?  *En el último año, ¿alguna vez se sintió insegura(o) en su hogar?* | □ Yes/Sí □ No |
| 5. In the past year, have you ever felt at risk for being homeless?  *En el último año, ¿alguna vez se sintió en riesgo de quedarse sin hogar?* | □ Yes/Sí □ No |
| 6. In the past year, have you ever had to go without health care because you didn’t have a way to get to the clinic?  *En el último año, ¿alguna vez estuvo sin atención médica porque no pudo llegar a la clínica?* | □ Yes/Sí □ No |
| 7. Does your family need a lawyer to help with your landlord, housing, immigration, or taxes?  *¿Su familia necesita a un abogado para ayudarle con el dueño de su vivienda, con vivienda, inmigración o impuestos?* | □ Yes/Sí □ No |
| 8. In the past year has the electric, gas, oil, or water company threatened to shut off services in your home?  *En el último año, ¿La empresa de servicios públicos la amenazó con cortarle los servicios de electricidad, gas, aceite o agua de su casa?* | □ Yes/Sí □ No |
| 9. As a caregiver, in the past year, have you often felt down, depressed, or hopeless?  *En el último año, ¿mientras cuidaba a su familia ¿se sintió con frecuencia disgustada(o), deprimida(o) o desesperanzada(o)?* | □ Yes/Sí □ No |
| 10. As a caregiver, in the past year, have you had a problem with drugs or alcohol?  *En el último año, ¿mientras cuidaba a su familia tuvo usted problemas con el abuso de alcohol o drogas?* | □ Yes/Sí □ No |
| 11. Are there any other problems you’d like help with today?  *¿Hay algún otro problema con el que necesite ayuda hoy?* | □ Yes/Sí □ No |

**Supplementary Table 2**: TRIPOD Checklist

| **Section/Topic** | **Item** |  | **Checklist Item** | **Page** |
| --- | --- | --- | --- | --- |
| **Title and abstract** | | | | |
| Title | 1 | D;V | Identify the study as developing and/or validating a multivariable prediction model, the target population, and the outcome to be predicted. | 1 |
| Abstract | 2 | D;V | Provide a summary of objectives, study design, setting, participants, sample size, predictors, outcome, statistical analysis, results, and conclusions. | 2 |
| **Introduction** | | | | |
| Background and objectives | 3a | D;V | Explain the medical context (including whether diagnostic or prognostic) and rationale for developing or validating the multivariable prediction model, including references to existing models. | 3-4 |
|  | 3b | D;V | Specify the objectives, including whether the study describes the development or validation of the model or both. | 3-4 |
| **Methods** | | | | |
| Source of data | 4a | D;V | Describe the study design or source of data (e.g., randomized trial, cohort, or registry data), separately for the development and validation data sets, if applicable. | 4 |
|  | 4b | D;V | Specify the key study dates, including start of accrual; end of accrual; and, if applicable, end of follow-up. | 4 |
| Participants | 5a | D;V | Specify key elements of the study setting (e.g., primary care, secondary care, general population) including number and location of centres. | 4-5 |
|  | 5b | D;V | Describe eligibility criteria for participants. | 4-5 |
|  | 5c | D;V | Give details of treatments received, if relevant. | N/A |
| Outcome | 6a | D;V | Clearly define the outcome that is predicted by the prediction model, including how and when assessed. | 5 |
|  | 6b | D;V | Report any actions to blind assessment of the outcome to be predicted. | N/A |
| Predictors | 7a | D;V | Clearly define all predictors used in developing or validating the multivariable prediction model, including how and when they were measured. | 6-7 |
|  | 7b | D;V | Report any actions to blind assessment of predictors for the outcome and other predictors. | N/A |
| Sample size | 8 | D;V | Explain how the study size was arrived at. | 8 |
| Missing data | 9 | D;V | Describe how missing data were handled (e.g., complete-case analysis, single imputation, multiple imputation) with details of any imputation method. | 9 |
| Statistical analysis methods | 10a | D | Describe how predictors were handled in the analyses. | 9 |
|  | 10b | D | Specify type of model, all model-building procedures (including any predictor selection), and method for internal validation. | 8-9 |
|  | 10c | V | For validation, describe how the predictions were calculated. | 8 |
|  | 10d | D;V | Specify all measures used to assess model performance and, if relevant, to compare multiple models. | 9 |
|  | 10e | V | Describe any model updating (e.g., recalibration) arising from the validation, if done. | N/A |
| Risk groups | 11 | D;V | Provide details on how risk groups were created, if done. | N/A |
| Development vs. validation | 12 | V | For validation, identify any differences from the development data in setting, eligibility criteria, outcome, and predictors. | 8 |
| **Results** | | | | |
| Participants | 13a | D;V | Describe the flow of participants through the study, including the number of participants with and without the outcome and, if applicable, a summary of the follow-up time. A diagram may be helpful. | 10 |
|  | 13b | D;V | Describe the characteristics of the participants (basic demographics, clinical features, available predictors), including the number of participants with missing data for predictors and outcome. | 10 |
|  | 13c | V | For validation, show a comparison with the development data of the distribution of important variables (demographics, predictors and outcome). | 18-21 |
| Model development | 14a | D | Specify the number of participants and outcome events in each analysis. | 10 |
|  | 14b | D | If done, report the unadjusted association between each candidate predictor and outcome. | Supp Tab 3 |
| Model specification | 15a | D | Present the full prediction model to allow predictions for individuals (i.e., all regression coefficients, and model intercept or baseline survival at a given time point). | 20 |
|  | 15b | D | Explain how to the use the prediction model. | 12 |
| Model performance | 16 | D;V | Report performance measures (with CIs) for the prediction model. | 11-12, 22-24 |
| Model-updating | 17 | V | If done, report the results from any model updating (i.e., model specification, model performance). | N/A |
| **Discussion** | | | | |
| Limitations | 18 | D;V | Discuss any limitations of the study (such as nonrepresentative sample, few events per predictor, missing data). | 14 |
| Interpretation | 19a | V | For validation, discuss the results with reference to performance in the development data, and any other validation data. | 10 |
|  | 19b | D;V | Give an overall interpretation of the results, considering objectives, limitations, results from similar studies, and other relevant evidence. | 10-14 |
| Implications | 20 | D;V | Discuss the potential clinical use of the model and implications for future research. | 13 |
| **Other information** | | | | |
| Supplementary information | 21 | D;V | Provide information about the availability of supplementary resources, such as study protocol, Web calculator, and data sets. | 6, 10, 11 |
| Funding | 22 | D;V | Give the source of funding and the role of the funders for the present study. | 1 |

*Items relevant only to the development of a prediction model are denoted by D, items relating solely to a validation of a prediction model are denoted by V, and items relating to both are denoted D;V. We recommend using the TRIPOD Checklist in conjunction with the TRIPOD Explanation and Elaboration document.

**Supplementary Table 3**: Top 20 variable importance values (or equivalent) for best performing models in the training set.

| **Feature** | **Gradient boosting (gain)** | **Random forest (impurity)** | **Logistic regression (odds ratio; p-value)** |
| --- | --- | --- | --- |
| dv | 0.292861132 | 60.9 | 0.24; <.0001 |
| prev_FI | 0.250972525 | 163.7 | 4.09; <.0001 |
| ageY | 0.125254335 | 398.7 | 0.95; <.0001 |
| MIN_EST_geo | 0.066318617 | 135.6 | 1.00; 0.0594 |
| BMI_PERCENTILE | 0.055725143 | 429.9 | 1.00; 0.0150 |
| POVERTY_EST_geo | 0.036854262 | 129.5 | 1.01; <.0001 |
| POP_ESTIMATE_geo | 0.032691344 | 124.6 | 1.00; 0.0121 |
| transp | 0.025390430 | 49.8 | 1.18; 0.0309 |
| UNEMPLOYED_EST_geo | 0.024852297 | 112.0 | 1.01; 0.0322 |
| INCOME_ESTIMATE_geo | 0.016828527 | 146.3 | 1.00; 0.7459 |
| EDU_EST_geo | 0.015285987 | 126.3 | 1.00; 0.9750 |
| TRANSPORT_EST_geo | 0.014298883 | 99.3 | 1.00; 0.1569 |
| female | 0.012351969 | 79.2 | 0.89; 0.0045 |
| HOUSE_ESTIMATE_geo | 0.010326198 | 118.1 | 1.00; 0.3920 |
| OWNERSHIP_EST_geo | 0.005760296 | 125.5 | 1.00; 0.6553 |
| wtF | 0.004022933 | 30.4 | 1.43; 0.0007 |
| preM | 0.003266379 | 29.3 | 1.24; 0.0264 |
| hosp | 0.002628289 | 16.7 | 1.13; 0.4007 |
| homeless | 0.002159283 | 54.0 | 1.08; 0.4997 |
| AGE_ESTIMATE_geo | 0.002151171 | 127.6 | 1.00; 0.9482 |

**Supplementary Table 4:** Confusion matrices for LR, RF, and GBM in test set. Note: cutpoint set to 0.5. ROC curves and subsequent table examine range of cutpoints.

|  | LR | | | RF | | | GBM | | |
| --- | --- | --- | --- | --- | --- | --- | --- | --- | --- |
|  | FI | No FI | Total | FI | No FI | Total | FI | No FI | Total |
| Predict FI | 18 | 29 | 47 | 25 | 49 | 74 | 46 | 71 | 117 |
| Predict No FI | 748 | 4186 | 4934 | 741 | 4166 | 4907 | 720 | 4144 | 4864 |
| Total | 766 | 4215 | 4981 | 766 | 4215 | 4981 | 766 | 4215 | 4981 |

**Supplementary Table 5:** DeLong’s test for the difference in AUC values resulted in the following findings. LR vs. GBM (p=0.01443), LR favored; RF vs. LR (p<.0001), LR favored; RF vs. GBM (p=.0102), GBM favored. The cutpoints offering the best value of sensitivity+specificity (Youden’s J statistic) are displayed.

|  | Cutpoint | Sensitivity | Specificity | PPV | NPV |
| --- | --- | --- | --- | --- | --- |
| LR | 0.1320 | 0.6076 | 0.6893 | 0.9150 | 0.2420 |
| RF | 0.1971 | 0.7566 | 0.4713 | 0.8873 | 0.2603 |
| GBM | 0.3995 | 0.7786 | 0.4935 | 0.8943 | 0.2883 |

Legend: PPV = positive predictive value. NPV = negative predictive value. LR = logistic regression. RF = random forest. GBM = gradient boosted model.

**Supplementary Table 6**: Results from 10-fold cross-validation on training set, by geographic level (census block vs. zip code) and lookback time (12, 18, 24 months). Missing data coded as indicator rather than imputed.

|  |  | **Census block** | | | **ZIP code** | | |
| --- | --- | --- | --- | --- | --- | --- | --- |
|  |  | **12** | **18** | **24** | **12** | **18** | **24** |
| **Accuracy** | LR | 0.8500 | 0.8496 | 0.8509 | 0.8512 | 0.8505 | 0.8502 |
|  | RF | 0.8491 | 0.8483 | 0.8473 | 0.8492 | 0.8497 | 0.8500 |
|  | GBM | 0.8487 | 0.8490 | 0.8476 | 0.8485 | 0.8497 | 0.8468 |
| **AUC** | LR | 0.6658 | 0.6812 | 0.6876 | 0.6596 | 0.6763 | 0.6826 |
|  | RF | 0.6559 | 0.6738 | 0.6714 | 0.6552 | 0.6704 | 0.6753 |
|  | GBM | 0.6412 | 0.6622 | 0.6680 | 0.6477 | 0.6650 | 0.6680 |
| **PR-AUC** | LR | 0.2981 | 0.3079 | 0.3095 | 0.2982 | 0.3048 | 0.3062 |
|  | RF | 0.2725 | 0.2772 | 0.2708 | 0.2877 | 0.2927 | 0.2949 |
|  | GBM | 0.2772 | 0.2856 | 0.2824 | 0.2814 | 0.2940 | 0.2878 |
| **Sensitivity** | LR | 0.0607 | 0.0536 | 0.0545 | 0.0639 | 0.0584 | 0.0518 |
|  | RF | 0.0415 | 0.0373 | 0.0322 | 0.0389 | 0.0383 | 0.0367 |
|  | GBM | 0.0622 | 0.0639 | 0.0624 | 0.0730 | 0.0696 | 0.0662 |
| **Specificity** | LR | 0.9905 | 0.9914 | 0.9928 | 0.9914 | 0.9916 | 0.9924 |
|  | RF | 0.9931 | 0.9929 | 0.9927 | 0.9935 | 0.9943 | 0.9948 |
|  | GBM | 0.9888 | 0.9888 | 0.9876 | 0.9865 | 0.9886 | 0.9860 |
| **PPV** | LR | 0.5202 | 0.5091 | 0.5596 | 0.5671 | 0.5570 | 0.5620 |
|  | RF | 0.5408 | 0.5042 | 0.4574 | 0.5145 | 0.5421 | 0.5555 |
|  | GBM | 0.4990 | 0.5000 | 0.4687 | 0.4844 | 0.5246 | 0.4615 |
| **NPV** | LR | 0.8555 | 0.8546 | 0.8549 | 0.8560 | 0.8553 | 0.8545 |
|  | RF | 0.8532 | 0.8526 | 0.8519 | 0.8530 | 0.8530 | 0.8529 |
|  | GBM | 0.8554 | 0.8557 | 0.8552 | 0.8566 | 0.8564 | 0.8556 |

Legend: AUC = area under the cure. PR-AUC = precision recall- area under the curve. PPV = positive predictive value. NPV = negative predictive value. LR = logistic regression. RF = random forest. GBM = gradient boosted model.

**Supplementary Table 7**: Model performance in test set. Missing data coded as indicator rather than imputed. Predictors chosen are those at the census block level and a 24 month look back, the best performing set in Supplementary Table 3.

|  | **Census block + 24 month lookback** | |
| --- | --- | --- |
| **Accuracy** | LR | 0.8466 |
|  | RF | 0.8464 |
|  | GBM | 0.8480 |
| **AUC** | LR | 0.7024 |
|  | RF | 0.6760 |
|  | GBM | 0.6816 |
| **PR-AUC** | LR | 0.3023 |
|  | RF | 0.2785 |
|  | GBM | 0.2971 |
| **Sensitivity** | LR | 0.0405 |
|  | RF | 0.0274 |
|  | GBM | 0.0392 |
| **Specificity** | LR | 0.9931 |
|  | RF | 0.9953 |
|  | GBM | 0.9950 |
| **PPV** | LR | 0.5167 |
|  | RF | 0.5122 |
|  | GBM | 0.5882 |
| **NPV** | LR | 0.8506 |
|  | RF | 0.8492 |
|  | GBM | 0.8507 |

Legend: PPV = positive predictive value. NPV = negative predictive value. LR = logistic regression. RF = random forest. GBM = gradient boosted model.
